# The Easter Bank Holiday, 1917

**Published:** 1917-04-14

**Authors:** 


### THE EASTER BANK HOLIDAY, 1917.

Few members of the present generation have more deeply appreciated the call for a holiday than those who have been continuously occupied, under war conditions, which involve long hours, frequently lengthening, with shortened periods of rest and no leisure worthy of the name. A benevolent Government, as Governments are apt to do, taking the line of least resistance and refusing to look ahead further than they were obliged, had announced that the Easter holiday in 1917 should extend without a break from the Thursday before Easter to the following Tuesday. The Government's name for this period of relaxation was a Bank Holiday, and not a general holiday, so that it is impossible to be sure how many and which sections of the public considered themselves included under this definition. The shopkeepers of most intelligence, having regard no doubt to business and their customers' convenience, opened on Saturday and extended the hours of opening longer than usual as an exception in exceptional circumstances.

The railway managers everywhere have persistently discouraged railway travelling, and especially travellers who must have luggage with them; but, despite this, the exodus from London on the first day was considerable, an exodus which called for courage. The unfavourable weather conditions prohibited the thought of a country holiday to most practical people, especially when they had to be fit to return to hard work in the pink of condition, whether they indulged in a four days' gamble with the weather or declined it. How far the exodus from the provinces to London proved appreciable, is not apparent. Many, we should be justified in saying most country residents, have had their thoughts turned to agricultural pursuits by the Minister in charge of this Department. The greater the patriotism the stronger has been the feeling that it was a duty to spend the holidays spade in hand, providing always the weather permitted the ground to be in a workable condition. We have had a pæan of rejoicing from one correspondent, not given to physical exertion, who has found spade labour and the companionship it has brought him both exhilarating and health-giving. He is a poet by instinct, and the spade and the sod have stirred his imagination, quickened his circulation, and made a man of him, to his great surprise and rejoicing. We are not without hope, therefore, that those whom the tribunals, conscientious objections, and the absence of a robust physique have deprived of the exhilaration and muscle-producing qualities of military training, through spade work may yet attain to a physical development worthy of a full-grown British citizen, whatever the status in life may be. Lest imperfect development and conscientious objecting refuse to run together we may add that our experience convinces us that the former, in the majority of cases, might be quickly changed to physical fitness under the drill-sergeant, continuous outdoor exercise, the awakening of new experiences, and the growth in stamina sure to follow a well-regulated routine.

Reverting to the brain worker proper, who has probably worked harder in wartime than ever before in his life, the call for an Easter holiday has been strenuous and insistent. The conditions under which we live during wartime forbade even the most casual, cheerfully to leave all arrangements for taking a holiday until the day of contemplated departure. Those who needed a week's preparation were turned back by one of the heaviest snowfalls which Londoners have experienced this season. The clerk of the weather, cheered apparently by the robustness he has gained from the persistent and biting east winds of March, has laid himself out to play with the hungry holiday-seeker by giving him hourly samples of snow, rain, and sunshine, on successive days immediately preceding Easter. We are of opinion that all who were able have been wise to defer a holiday from home, rather than face the conditions of the country, where walking offered many disabilities, and where the birds, the flowers, and Nature generally were still suffering from the effects of an unusually severe winter, which has so far taken most of the joy from country life. We await with hope the spring days in the sunshine when everything combines to inspirit and cheer and life in the country makes men and women young and sprightly. Meanwhile we can cultivate the cheerful countenance, remembering that holidays deferred prove often joyous.

